# On Some Pathological Problems of To-Day

**Published:** 1900-08-18

**Authors:** Burdon Sanderson


### Hospital Clinics and Medical Progress.

### ON SOME PATHOLOGICAL PROBLEMS OF TO-DAY.

Abstract of an Address delivered before the Second General Assembly of the International Medical Congress in Paris, by Professor Burdon Sanderson, M.D., F.R.S.

Forty-five years ago, after having received the degree of Doctor of Medicine, I came to Paris to finish my medical education. Now that I have nearly arrived at the end of my medical and scientific career, you have done me the honour to invite me to address you, and while I desire to thank you most cordially for the invitation, I beg, at the same time, to express my gratitude to my French colleagues for the friendly reception they have given me.

After giving a sketch of the history of pathological investigation from the time of the enunciation of the doctrine of "cellular pathology," up to the commencement of what one may call the bacteriological period, Professor Burdon Sanderson said: "It is evident that the tendencies of pathological research have undergone a very marked change. In former times it was the histological characters of the processes, such as inflammation, tuberculosis, &c., which more especially interested us; now it is the microbe which attracts attention." This, however, is to be noted, that, although everyone recognises the enormous value of bacteriological diagnosis, and of the therapeutic uses of the serums, it is not in these directions that micro-biology has exercised its greatest influence on the science of medicine. Its influence has consisted rather in the new and wider idea which it has given us of the physiological functions of the cell, and consequently of cellular pathology. "Pathology is no less fundamentally 'cellular pathology' to-day than it was at the period when Virchow adopted this expression."

But if we still use the expression it is necessary to give to it a more extended signification than it then had, for we must include not only the histological changes, but the chemical reactions which take place between the cell and the medium in the midst of which it lives. In days gone by the notion that each kind of cell possessed a special function appeared to suffice. Now, however, it is necessary to consider their chemical relations. One recognises now that the leucocytes, which used to interest us chiefly by the part they bore in the processes of inflammation, and as types of contractile and amœboid protoplasm, possess special chemical functions; functions indispensable to the life of the organism.

The red blood cell also, which one used to regard only as the vehicle of hæmoglobin, has chemical susceptibilities which render it the most delicate test of abnormal conditions of the blood. The lymphoid cell, which is, so to say, all nucleus, gives to the blood products which differ from other cells, in that they are derived from the decomposition of nuclear substance. Does there then exist any general theory to which one can refer, and by which one can explain all these facts? The processes in which all these different kinds of cells take part have this in common that they resolve themselves into chemical reactions, and as the result of the researches of recent years one is led to believe that all these reactions—action of the cell on its surroundings, action of the surroundings on the cell—are the work of soluble ferments, which in virtue of their origin one may regard, to use the expression of Kühne, as enzymes—that is to say, ferments which are the products of the evolution of the living cell. May one, then, consider that all the normal functions of the cell are carried on by means of ferments? With the exception of the functions which are proper to the nervous system, I believe that one can give an affirmative answer to this question. The studies which have been made during the last few years on the ferments of the specific microbes allow us to attribute to their constituent ferments many of the chemical functions which one used to regard as belonging specially to the cell itself. Thus in the domain of micro-biology the enzymes have, so to say, "dethroned the cell." If we can extract from the cell one substance which respires for it, transmitting the oxygen from the air to the molecule of the oxydisable substance, and another which digests for it, which builds up complex from simple molecules, or undoes the complex molecules into the more simple—if that be so, the cell can no longer be considered the unit, as it was in times past. It is rather a chemical factory, where a great number of processes go on, which, according to the universal law of adaptation, contribute to maintain or to protect not only the individual cell, but the whole organism. If, then, one asks whether by so delegating the functions of the cell to its enzymes we advance our knowledge of the processes of life, one may answer that the only way of understanding or explaining vital phenomena is to determine the physical conditions under which they are produced. The essence of physiological experimentation consists in the elimination of the mystery of life. The first step is to reproduce "in vitro" what one has observed in life; for even when one understands neither the constitution nor the mode of action of the ferment, which is still the case with the greater number of the zymotic processes, one can study its relation to external conditions independently of its vital origin. One must not imagine, however, that a vital process of necessity becomes intelligible directly we have imitated it in the laboratory. Many things which we see are quite as incomprehensible as ever, but at least we are able to reproduce them at will, and to study and control the conditions under which they happen. Having got so far sooner or later we shall know the cause. When we can do the thing "in vitro" we as biologists have got as far as possible; the rest is for the chemists. We must be allowed, then, to take the theory of ferments and apply it as our useful guide for the discovery of new methods for analysing the chemical functions of the cell, always remembering that we must guard ourselves from giving it the authority of a biological law.

After stating that among the most exact experiments illustrating the multiplicity of the chemical functions of the animal cell are those which refer to the isolated cells, such as the red blood cells and the leucocytes, Professor Sanderson entered upon an account of the various reactions of these cells, and pointed out, in conclusion, that, although it might be impossible of demonstration, we must admit that in certain morbid conditions there exist between cells of varied origin, tissue cells in fact, relations which resemble those which it has been possible to study with such exactitude in regard to isolated cells such as the blood corpuscles. It is, he said, in the chemical functions of the cells that one must seek for the immediate and determining causes of morbid conditions, and if we desire to understand them we must continue to be, as before, "cellular pathologists." It is the application to the cells of the organisms of the same methods which have been employed with such success in the study of the chemical functions of the microbes, of the leucocytes, and the red blood cells, that offers to us the best hope of progress in the study of the pathology of organic life.

